# Translational Application of Circulating DNA in Oncology: Review of the Last Decades Achievements

**DOI:** 10.3390/cells8101251

**Published:** 2019-10-14

**Authors:** Natalia O. Tuaeva, Luca Falzone, Yuri B. Porozov, Alexander E. Nosyrev, Vladimir M. Trukhan, Leda Kovatsi, Demetrios A. Spandidos, Nikolaos Drakoulis, Alexandra Kalogeraki, Charalampos Mamoulakis, George Tzanakakis, Massimo Libra, Aristides Tsatsakis

**Affiliations:** 1 I.M. Sechenov First Moscow State Medical University (Sechenov University), 119048 Moscow, Russia; natalya.tuaeva@gmail.com (N.O.T.); yuri.porozov@gmail.com (Y.B.P.); rerik2050@mail.ru (A.E.N.); vladimir.trukhan@gmai.com (V.M.T.); tsatsaka@uoc.gr (A.T.); 2 Department of Biomedical and Biotechnlogical Sciences, University of Catania, 95123 Catania, Italy; 3 Epidemiology Unit, IRCCS Istituto Nazionale Tumori “Fondazione G. Pascale”, 80131 Naples, Italy; 4 ITMO University, Saint Petersburg 197101, Russia; 5 Laboratory of Forensic Medicine and Toxicology, School of Medicine, Aristotle University of Thessaloniki, 54248 Thessaloniki, Greece; kovatsi@med.auth.gr; 6 Laboratory of Clinical Virology, Medical School, University of Crete, Heraklion, 70013 Crete, Greece; spandidos@spandidos.gr; 7 Research Group of Clinical Pharmacology and Pharmacogenomics, Faculty of Pharmacy, School of Health Sciences, National and Kapodistrian University of Athens, 15771 Zografou, Greece; drakoulis@pharm.uoa.gr; 8 Department of Pathology-Cytopathology, Medical School, University of Crete, Heraklion, 70013 Crete, Greece; kalogerakimed@yahoo.gr; 9 Department of Urology, University General Hospital of Heraklion, University of Crete, Medical School, Heraklion, 70013 Crete, Greece; mamoulak@uoc.gr; 10 Laboratory of Anatomy-Histology-Embryology, Medical School, University of Crete, Heraklion, 70013 Crete, Greece; tzanakak@med.uoc.gr; 11 Research Center for Prevention, Diagnosis and Treatment of Cancer, University of Catania, 95123 Catania, Italy; 12 Department of Forensic Sciences and Toxicology, Faculty of Medicine, University of Crete, Heraklion, 71003 Crete, Greece

**Keywords:** ctDNA, biomarker, oncology, liquid biopsy, NGS, mass-spectrometry, glioma, urological cancers, diagnosis, prognosis

## Abstract

In recent years, the introduction of new molecular techniques in experimental and clinical settings has allowed researchers and clinicians to propose circulating-tumor DNA (ctDNA) analysis and liquid biopsy as novel promising strategies for the early diagnosis of cancer and for the definition of patients’ prognosis. It was widely demonstrated that through the non-invasive analysis of ctDNA, it is possible to identify and characterize the mutational status of tumors while avoiding invasive diagnostic strategies. Although a number of studies on ctDNA in patients’ samples significantly contributed to the improvement of oncology practice, some investigations generated conflicting data about the diagnostic and prognostic significance of ctDNA. Hence, to highlight the relevant achievements obtained so far in this field, a clearer description of the current methodologies used, as well as the obtained results, are strongly needed. On these bases, this review discusses the most relevant studies on ctDNA analysis in cancer, as well as the future directions and applications of liquid biopsy. In particular, special attention was paid to the early diagnosis of primary cancer, to the diagnosis of tumors with an unknown primary location, and finally to the prognosis of cancer patients. Furthermore, the current limitations of ctDNA-based approaches and possible strategies to overcome these limitations are presented.

## 1. Introduction

It has been widely demonstrated that several physiological and pathological conditions induce cells and tissues remodeling and, in turn, the rearrangement of stroma and tissue microenvironment. These events are generally sustained by necrotic or apoptotic processes, leading to the disaggregation of tissues and the consequent dissemination of cells and cellular debris in the intercellular space and in the bloodstream. Through these processes, circulating cells and DNA fragments reach the biological fluids and can be easily detected using different molecular techniques [1,2,3]. Other studies demonstrated that cells actively release cellular DNA in the extracellular space via the secretion of vesicles and exosomes, independently from cell necrosis or apoptosis [4,5]. This extracellular fraction of DNA is commonly called circulating free DNA (cfDNA), as the DNA fragments can be found in the biological fluids. The general term “cfDNA” encloses different types of circulating DNA, including cell-free DNA (cfDNA), cell-free fetal DNA (cffDNA), and circulating tumor DNA (ctDNA) [6].

Circulating tumor DNA is a tumor-derived fragmented DNA found in the bloodstream and other biological fluids, not associated with cfDNA which, on the contrary, is released by normal cells [7]. In the last decades, a growing body of evidence has defined the potential clinical value of ctDNA because it may recapitulate the entire tumor molecular profile. Therefore, it could be used for diagnostic, prognostic, monitoring, and therapeutic purposes [8].

**Figure 1:**
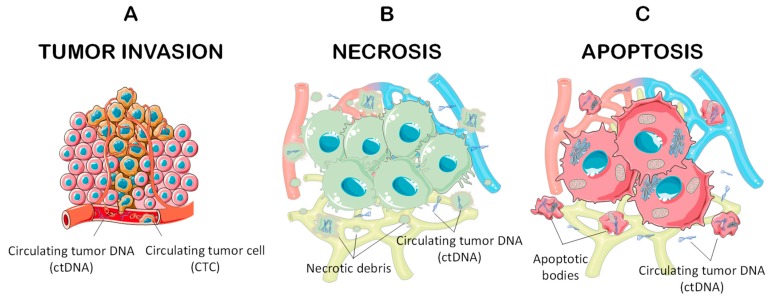
Release modalities of circulating tumor DNA (ctDNA) and circulating tumor cells (CTCs). (**A**) Invasive tumors release CTCs and ctDNA directly in the tumor infiltrating vessels or in invaded arteries and veins; (**B**) Necrotic cells release ctDNA directly into the cellular interstitium that reach the bloodstream through the lymphatic vessels; (**C**) Similarly, apoptotic cells release ctDNA directly in the cellular interstitium or through apoptotic bodies.

Several mechanisms have been described to explain the mechanism of ctDNA release in the bloodstream or into other body fluids [2]. The most accredited hypotheses involve either the release of DNA from the necrotic cells of the primary tumor bulk or the loosening of DNA derived from the lysis of circulating tumor cells (CTC) in the bloodstream and lymphatic vessels [2]. In particular, several studies have hypothesized that ctDNA release is the combination of various biological processes, including apoptosis, necrosis, and tumor invasion [1,9,10] (Figure 1). Furthermore, specific tumor features, i.e., the tumor localization, vascularization, and molecular characteristics, including loss of adhesion molecules (E-Cadherin, integrins, selectins, etc.), also facilitate the release of ctDNA and CTCs [11,12].

Recent studies on circulating extracellular DNA in human biological fluids (blood plasma, serum, urine, etc.) were performed with the final goal of improving oncology practice. Circulating tumor DNA, as well as genomic and epigenetic alterations, such as single nucleotide polymorphisms (SNPs) [13], chromosome rearrangements, copy number variation (CNV) [14,15], microsatellite instability (MSI) [16], loss of heterozygosity (LOH) [17], microRNA alterations [18], and specific changes in DNA methylation patterns [19,20,21], have been widely explored. Potentially, circulating DNA is the carrier of the genetic information deriving from the primary cells or tissues [22,23,24]. For example, a circulating DNA methylation profile may mirror the tumor localization, as each tissue has a specific methylation pattern. Furthermore, the DNA methylation profile may also provide information about the nucleosome positioning of the DNA sequence [24]. In general, transcription factor footprints may reflect the epigenetic cellular landscape, and hence the tissue origin of a certain ctDNA. Thus, they may be used for the identification of the corresponding unknown primary cancer [22,23].

**Figure 2:**
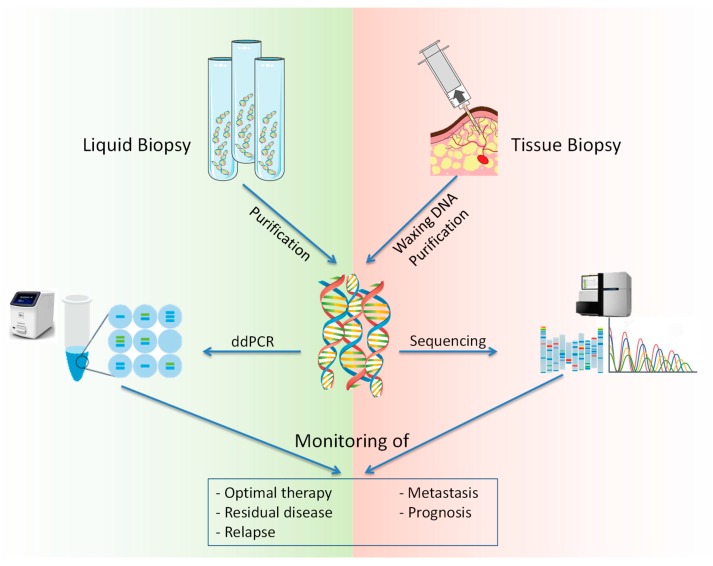
Comparison between liquid biopsy and tissue biopsy. The purification and/or extraction of DNA from liquid or tissue biopsy allows the researcher to perform high-throughput molecular analysis (Next Generation Sequencing or droplet digital PCR) in order to obtain significant data to define the prognosis of patients, to monitor the therapeutic efficacy and to predict the development of metastasis and relapse. Therefore, similarly to tissue biopsy, liquid biopsy allows the clinicians to obtain informative clinical data with a less invasive and less expensive method.

Recently, liquid biopsy, a new analytical approach based on the analysis of a peripheral blood sample for the identification of tumor-specific alterations directly in the bloodstream, was introduced in the clinical practice for diagnostic and prognostic purposes [25,26]. The goal of this approach was to replace tissue biopsy with minimally invasive techniques, simultaneously obtaining a large amount of DNA [27]. Indeed, it was widely proven that it is possible to recover cfDNA up to 100 ng/mL for healthy individuals and up to 1000 ng/mL for cancer patients from liquid biopsy samples [28]. Furthermore, the benefits of using the liquid biopsy are the high specificity and efficiency in monitoring tumor changes or disease progression. In this context, several studies demonstrated that liquid biopsy is able to spot precise circulating DNA mutations directly associated to a specific neoplasm [29,30]. This is possible because the short half-life of ctDNA (spanning between 16 min and 13 h) allows immediate correlation with tumor cell status and thus offers the possibility of a continuous dynamic observation [31,32,33,34]. In contrast, the classic tissue biopsy provides only a single space-time snapshot and does not reflect on the dynamic heterogeneity of the tumor. Plasma/serum-derived ctDNA samples do not degrade until analysis, while tissue preparations are obligatory fixed, either in formalin or in paraffin, which creates a risk of DNA cross-linking and fragmentation, thus violating the DNA’s structural integrity and interfering with its sequencing [35]. In detail, only 5 × 10^7^ tumor cells are necessary to obtain detectable amounts of ctDNA, and 10^9^ cells are necessary to obtain an image using high–resolution computer tomography [9]. This represents only one of the advantages of liquid biopsy in contrast to tissue biopsy (Table 1) (Figure 2).

Consequently, ctDNA levels in plasma or serum may offer early diagnosis in the very few months of disease progression, even before any detectable change in the tumor images obtained through X-Ray, MRI, PET, CT, or PET/CT or changes in blood protein markers levels [37,38]. In this context, several studies demonstrated that the analysis of ctDNA levels during anticancer treatments can be used in combination with the above mentioned imaging techniques in order to perform a more comprehensive evaluation of the tumor progression, thus optimizing the monitoring procedures of tumor [39,40,41]. The introduction of ctDNA analysis in clinical practice could indicate when to perform imaging diagnostic procedures to physicians, reducing the patients’ exposure to radiation and predicting inefficient treatments [42,43]. Nevertheless, still today ctDNA analysis cannot completely replace the above-mentioned imaging techniques.

It is clear that ctDNA levels correlate well with tumor size (tumor load) [38] and sharply decrease after resection [37]. Thus, liquid biopsy is a promising tool for detecting residual tumors, metastases, tumor progression or regression during therapy, or disease prognosis [21,34]. However, besides these advantages, the liquid biopsy may present some limitations, mainly related to the specific and highly sensitive instruments necessary to determine the expression levels of small quantities of ctDNA circulating in the peripheral blood [44].

This review discusses the most advanced applications of ctDNA analysis which have been developed in the last decade, listed in Table 2 (Table 2).

## 2. NGS, ddPCR, and Liquid Biopsy for ctDNA Analysis

It is well-accepted the notion that early diagnosis of tumor is a key factor in the management of cancer patients, which is essential to enhance the overall survival and progression-free survival of patients [63]. The main objective of early diagnosis is to detect symptomatic patients as early as possible in order to avoid delays in the administration of anticancer treatments.

The concept of “early diagnosis” should not be confused with “screening,” the former consisting in the identification of a disease in healthy and asymptomatic patients through the use of tests and/or medical procedures applied to specific population at risk for such disease [64].

Compared to early diagnosis, cancer screening consists of a specific investigative strategy encompassing a wider geographical area and is directed toward a target population. In this context, several screening programs have been developed for the early identification of different types of cancers [65]. Both early diagnosis and screening programs were effective in reducing cancer deaths [66].

In the last decade, greatest attention has been paid to the use of liquid biopsy as a good screening method for both hematological and solid tumors, as well as its role as a new diagnostic test available for the early diagnosis of cancer [67].

In this context, the development of novel next generation sequencing (NGS) methods has made a significant contribution to the early diagnosis of cancer based on the detection of low-frequency mutations in ctDNA [68]. As known, NGS is a high-throughput technology able to sequence the whole genome at once. In recent years, different types of NGS approaches have been developed. All these techniques are generally characterized by the fragmentation of the genomic DNA into small sequences, which are subsequently amplified in order to perform the automated sequencing of multiple fragments at once (the so-called massively parallel sequencing) [69]. Nowadays, NGS is applied to several analyses, including ctDNA sequencing.

NGS allows the detection of ctDNA changes occurring in the whole genome [70,71,72], thus providing an opportunity to comprehensively profile tumor-related genetic changes [73,74]. This is important to have the possibility to detect cancer clones able to mutate rapidly and, therefore, to become resistant to specific anticancer drugs [74]. Target diagnostic panels of tumor-specific mutations (“hot spots”) in ctDNA, including the most characteristic mutations of proto-oncogenes and suppressors, are being developed for primary preclinical diagnosis based on NGS [45]. Several types of such platforms are currently in use, including tagged-amplicon deep sequencing (TAmSeq) [31,45,75], Ion-AmpliSeq (Thermo Fisher Scientific) [76,77,78], and molecular cluster sequencing of Illumina [46]. Indeed, Forshew et al. identified mutations in the onco-suppressor gene TP53 of ctDNA in plasma samples of 46 patients with progressive ovarian cancer with a sensitivity and specificity greater than 97%, whereas the frequency of the mutant allele occurrence in the circulating DNA in plasma samples was no more than 2% using TAm-Seq [45]. In a separate study, the authors identified a particular mutation of the EGFR gene, not detected in the primary ovarian biopsy, in plasma circulating DNA. Using the same technology, these authors monitored the tumor dynamics through the estimation of 10 concomitant mutations in patients with metastatic breast cancer [45]. Moreover, Hi-Seq (Illumina) sequencing of the five “hot points” (*KRAS, TP53, APC, FBXW7, SMAD4*) in ctDNA from 26 patients with pancreatobiliary carcinoma was carried out with a diagnostic accuracy of 97.7%, sensitivity of 92.3%, and specificity of 100% [46]. 

Recently, an exceptionally promising method (CancerSEEK) was developed for the primary screening of eight types of cancer (ovaries, liver, stomach, pancreas, esophagus, rectum, lungs, and breast), which focuses on a small panel of markers (16 genes and eight proteins) and is based on massively parallel sequencing [36]. The authors emphasized that the method is intended for screening, and therefore differs from other molecular tests based on the analysis of a large number of genes with the aim of identifying therapeutic targets. The small panel may reduce false positive results and the elevated costs of a larger analysis [36,79]. The screening was performed on 1005 patients with metastatic tumors and 812 healthy individuals, showing only 1% of false positive results, 70% median sensitivity, and ≥99% specificity [36].

The GRAIL study, launched by Illumina to develop its preclinical cancer detection technology in blood by high-intensity sequencing assays, population-scale clinical studies, and machine learning such as neural networks, started in 2016 and will last five years. The GRAIL Clinical Research Program set up is presented in Supplementary Table S1. Thus, we can expect to have results soon, which will allow the development of a method for early cancer diagnosis in 2020–2021 (Table S1).

Next-generation sequencing (NGS) platforms are not the only tools available for the efficient analysis of ctDNA samples. In the last years, the digital PCR, a new type of polymerase chain reaction, was developed and efficiently used for the detection of small amount of ctDNA [80]. Among the different types of digital PCRs, the most promising is the droplet digital PCR (ddPCR) technique, which ensures high levels of sensitivity, as well as specificity (1:10,000 copies of DNA). Several studies demonstrated that ddPCR allows the identification of mutated DNA in peripheral blood and other liquid matrices with a high grade of accuracy, thus representing one of the most effective methods to analyze liquid biopsy samples for diagnostic purposes [81,82].

McEvoy and coworkers (2018) analyzed the circulating-free DNA samples of 32 melanoma patients and showed that ddPCR was able to detect BRAF, NRAS, or KIT mutations in all the patients with a mutational tumor burden >10 (23 out of 32) [83]. Similarly, a recent meta-analysis of 11 non-small cell lung carcinoma (NSCLC) studies showed that ddPCR displays a good performance for the detection of EGFR T790M mutation in ctDNA samples from a total of 872 advanced NSCLC patients. In particular, the overall concordance between plasma and tissue estimation was 81.2%, while the pooled analysis revealed that ddPCR test performs with 86.9% (95% CI, 80.6%–91.7%) of specificity and 70.1% (95% CI, 62.7%–76.7%) of sensitivity in detecting the T790M EGFR mutation in ctDNA samples from NSCLC patients [84]. Other similar studies were performed involving oral cancer patients [85], breast cancer patients [86], chronic myelogenous leukemia patients [87], and others.

Overall, both the NGS and the ddPCR methodology may be used for the effective detection of ctDNA. The choice of the best platform depends on the molecular target examined. The NGS is used to carry out a wide screening of the possible circulating mutations associated with the tumor, whereas the ddPCR is generally used for the identification of specific tumor mutations. 

## 3. Epigenetic Modifications of Circulating DNA May Reflect Tissue Origin of an Unknown Primary Cancer

It was widely demonstrated that a wide range of substances, including pesticides, pollutants, fibers, and heavy metals, might induce genetic and epigenetic damages to the cells predisposing for a plethora of diseases, including cancer [88,89,90,91,92]. Other physio-pathological processes, such as infections, chronic inflammation, and microbiota dysbiosis, have been also associated to the development of cancer [93,94,95,96]. 

In this context, the recent theory of real life risk simulation (RLRS) demonstrated that all the aforementioned risk factors interact with each other, highlighting a real hazard for human health [97,98,99]. According to this theory, demonstrated both in animal and human models [100,101,102], cumulative and synergistic effects of various seemingly harmless substances can be highlighted or even unveiled as leading mechanisms of epigenetic alterations and, in turn, the development of neurological disorders, cancers, and other diseases [103]. 

It is clear that all these environmental factors are able to induce the development of cancers through several molecular modifications directly detectable in ctDNA samples. A growing body of evidence reported that the epigenetic changes induced by all these mechanisms might predict the presence of diseases, as well as the aboriginal tissue. In this way, it is possible to determine the precancerous or cancerous tissue of origin, thus identifying cancers with unknown primary localization whose frequency is 4–5% of all registered invasive cancer types [104]. 

Circulating DNA may contain genetic features of cells derived from tissue from other individuals, thus its analysis is widely used as a non-invasive prenatal diagnosis and for predicting the risk of host versus graft disease in transplant medicine [105]. In both cases, DNA differences between two different organisms provide significant assistance in their identification. The epigenetic differences allow the identification of the tissue where the circulating DNA is derived from. In fact, it has previously been demonstrated that the distance between nucleosomes varies among cell types depending on the state of chromatin and gene expression [106,107,108]. Snyder (2016) demonstrated that deep sequencing of circulating DNA in plasma allows the generation of nucleosome location maps. The obtained maps correlated well with the architecture of the nucleus, genes structure, as well as their cellular expression [22]. Furthermore, it was demonstrated that small fragments of circulating DNA contain traces of nuclear transcription factors. Indeed, DNAse I fragmentation, used to study the tissue origin of circulating DNA, showed that hematopoietic cells have a large proportion of fragments with an extended distance between the nucleosomes due to the transcription factors mediated DNAse I-sensitive (DNS) sites shielding. The transcription start site (TSS) protection patterns are also quite different among various cell types. The epigenetic characteristics of circulating DNA in healthy humans indicate that it originates mainly from lymphoid and myeloid cells due to their physiological short lifespan. Furthermore, five patients suffering from various stage IV cancers (small cell lung cancer, squamous cell lung cancer, colorectal adenocarcinoma (CRC), hepatocellular carcinoma, and ductal carcinoma of the mammary gland in situ) showed ctDNA-derived nucleosome spacing patterns overlapping with relevant reference maps from corresponding cancer cell lines. For example, ctDNA from a patient with hepatocellular carcinoma showed the highest correlation with the HepG2 cell line (cell line for hepatocellular carcinoma), and the tumor ctDNA of breast duct carcinoma corresponded to MCF-7 (metastatic adenocarcinoma cell line). The limitation of this study was the small number of samples studied (n = 8) and the relatively small size of the reference data set of cell lines and tissues used for comparison (n = 76) [22]. For a full assessment of the potential and limitations of this approach, it is necessary to increase the number of samples studied, as well as the range of physiological states and diseases related to these samples.

Besides nucleosome modification, other epigenetic alterations are widely described in literature and might be responsible for cancer development and progression [109]. All these epigenetic changes can be detected through the analysis of ctDNA or other circulating elements (miRNAs, lncRNA, exosomes, etc.). As described above, environmental factors may induce molecular alterations in the DNA structure or may lead to the expression of non-coding RNAs (ncRNAs) which are, in turn, able to modulate the expression of several genes involved in cancer development [110,111]. In the first case, the most studied and common DNA backbone modification is the methylation occurring in the cytosine of CG dinucleotides (CpG site), able to modulate gene expression [112]. With respect to the modulation of non-coding RNAs or other elements, such as exosomes, different studies demonstrated that epigenetic changes may induce their strong deregulation, thus favoring the development and progression of cancer [18,113,114].

Both these epigenetic modulations, i.e., DNA methylation and alterations of the expression levels of ncRNA, can be effectively studied through liquid biopsy and the analysis of circulating nucleic acids [115,116].

Liang W. and colleagues developed recently a blood-based test for the detection of early-stage lung cancer using a non-invasive and high-sensitive high-throughput DNA bisulfite sequencing test. In particular, through the sequencing of more than 250 tissue samples, the authors identified nine methylation markers, achieving 79.5% (63.5%–90.7%) of sensitivity and 85.2% (66.3%–95.8%) of specificity when these markers were tested in a validation cohort of circulating DNA obtained from lung cancer and healthy patients [115].

Similarly, Menschikowski M and coworkers developed an optimized bias-based pre-amplification-digital droplet PCR (OBBPA-ddPCR) for the detection of methylated tumor DNA fragments levels with the goal of developing a diagnostic tool for early prostate cancer diagnosis. In particular, the authors showed that as few as five copies of methylated ctDNA out of 700,000 copies of unmethylated ctDNA fragments were identified through the use of ddPCR pre-amplification. Using this technique for the analysis of serum samples derived from 22 prostate cancer patients and 18 normal individuals, the authors reported a specificity of 100% in the detection of specific methylated markers [117].

Considering the analysis of ncRNA as indicator of epigenetics modification in cancer, several studies demonstrated the utility of liquid biopsy for the analysis of miRNAs and lncRNAs expression levels. In a recent review article, Pardini B. reported the state of the art of miRNAs analysis in several cancer types by using liquid biopsy. The authors showed that specific sets of miRNAs were successfully identified for almost all solid and hematological cancers [118]. In the same manner, the authors described the important achievements regarding the analysis of other circulating biomarkers in liquid biopsy samples, including piRNAs, snRNAs and snoRNAs, circRNA and lncRNA [118].

## 4. Circulating DNA as a Prognostic Criterion in Oncology

The prognostic properties of circulating DNA in oncology could help in choosing the most appropriate therapeutic approach (for example, in the case of drug resistance), thus avoiding ineffective treatment methods, as well as unwanted side effects and associated costs [119]. Nevertheless, ctDNA analysis for the prognosis of disease progression remains controversial. For example, Huang Z.H. et al. analyzed the correlation between plasma levels of and clinical-pathological parameters in breast cancer patients. Although circulating DNA concentrations were higher in patients with advanced cancer than in controls, there was no statistically significant difference [120]. In contrast, in patients with kidney carcinoma treated with sorafenib, higher ctDNA levels correlated with poorer prognosis [121], or predicted postoperative relapse with high sensitivity (91%) and specificity (100%) [122]. The absence of significant correlation of the total circulating DNA pool with prognosis may reflect the presence of other DNA sources, which may be associated with tumor growth (for example, apoptosis or necrosis of the adjacent tissue cells). 

A 2015 study focusing on predictive value comparison of ctDNA pool levels and circulating tumor cells revealed that the ctDNA concentrations neither reflected disease prognosis nor survival, although the number of circulating cells slightly correlated with both overall survival (OS) and progression-free survival (PFS) time [123]. The interference of separate factors, such as the individual activity of blood nucleases or enhanced phagocytosis, which, in some cases, leads to paradoxically low levels of ctDNA in patients with progressive disease, is also possible. Indeed, this was observed in several metastatic breast cancer patients [124] and two patients with bladder carcinoma [125]. The quantitative indicator is unlikely to be informative for predicting the patients’ outcome and it is necessary for future efforts to focus on specific DNA mutations and polymorphisms.

These data show that the analysis of ctDNA for prognostic purposes may be valid only for some tumors. In this context, concordant data were generated for some cancers, such as cutaneous melanoma, NSCLC, and colorectal cancer, where the evaluation of ctDNA before the beginning or during pharmacological treatments provided useful information to define patients’ OS and PFS [126,127,128,129]. Moreover, encouraging evidence shows that the evaluation of ctDNA or circulating tumor cells is useful to predict the response rate of patients to anti-tumor treatments, including recent immunotherapy. In this regard, in NSCLC and melanoma patients with advanced tumors, it was demonstrated that the evaluation of different molecular factors contained in liquid biopsy samples gave accurate information for the prediction of treatment outcomes [130]. The NGS evaluation of tumor mutational burden in NSCLC plasma samples revealed that patients with more than six ctDNA genetic alterations had higher response rates when treated with immune checkpoint inhibitors [131] and that more than 10 genetic alterations were associated with a prolonged PFS [132]. 

Other studies demonstrated that the initial increase of ctDNA percentages, followed by a drastic reduction of the number of mutated DNA copies, is associated to a good clinical response and therefore to a better prognosis. On the contrary, the initial reduction of mutated DNA, followed by a new increase of ctDNA, is associated to therapeutic failure and the onset of drug resistance mechanisms [130,133].

Thus, in addition to several ongoing studies, these data demonstrate that the analysis of ctDNA and liquid biopsy samples obtained during the treatment may give important information capable of monitoring the prognosis of patients, as well as identifying specific therapeutic protocols pursuing the principles of personalized medicine.

It is important to note that differences in both ctDNA amount and CTCs number could be linked to the pathological stage of tumor. In particular, a recent study performed on early and metastatic breast cancer patients showed significant differences both in CTCs and ctDNA detectable levels in early versus metastatic tumors for PIK3CA, E545K, and H1047R hotspot mutations [134]. Additionally, Tzanikou E. and colleagues demonstrated that such mutations were present in 39% of early cancer ctDNA plasma samples and in 47.9% of metastatic cancer ctDNA plasma samples. Similarly, the analysis of CTCs showed that the 48.2% of early cancer patients and the 66.6% of metastatic breast cancer patients presented the analyzed PIK3CA mutations, not detected in the healthy controls [134]. Taken together, these data showed that the analysis of CTCs was more sensitive compared to ctDNA analysis for the identification of PIK3CA mutations in liquid biopsy samples obtained from breast cancer patients.

Other studies demonstrated that the concomitant evaluation of CTCs and cfDNA can be used for an effective cancer prognosis. Marie-Hélène Delfau-Larue and colleagues reported that CTCs and bcl2-JH mutation detected in cfDNA might predict the disease progression, reflecting the tumor burden [135]. In particular, the researchers demonstrated that the analysis of both CTCs and cfDNA in follicular lymphoma patients at early-, mid-, and late-stages might give important information for a correct prognosis. Importantly, both CTCs and cfDNA correlate with total metabolic tumor volume (TMTV). However, only the number of bcl2-JH mutated copies detected in cfDNA adds extra prognostic information to the TMTV evaluation [135].

Although ctDNA analysis has been shown to be useful for both early diagnosis of tumors and to define cancer patients’ prognosis, the analysis of CTCs appears to be more suitable for monitoring patients with medium or advanced stage of the tumor in order to establish the overall survival and the progression free survival. Starting from these considerations, the research group coordinated by Rossi G. recently performed a study on metastatic breast cancer patients evaluating both CTCs and ctDNA [136]. In this study, the authors confirmed that CTCs levels at baseline are predictors of both PFS and OS in metastatic breast cancer setting, with a cutoff level of five cells per 7.5 mL of the whole blood. The authors further demonstrated that ctDNA correlated with the prognosis of patients and the tumor burden with a concordant rate (ranging between 72.5% and 100%) in the identification of mutations observed in tissue biopsies [136].

Overall, these studies showed that the presence of ctDNA or CTCs is associated with the presence of tumors, even early-stage tumors, while higher amounts of ctDNA and/or CTCs are correlated with higher risk of disease progression and with a poorer prognosis for the patients, independently from their tumor stage.

## 5. Circulating Tumor DNA Analysis for Cancer Personalized Medicine

A personalized approach in cancer diagnosis implies integral tumor profiling for each individual patient, which might be possible by tracking plasma ctDNA tumor-related mutations. The purpose of studying biopsy specimens maybe the selection of a personalized anticancer therapy, relevant to the mutational profile of the specific tumor. Application of the plasma ctDNA analysis allows for the monitoring of disease dynamics and the prescribed therapy effectiveness in order to detect any residual tumor after resection, relapse, or even metastasis within a particular patient. 

Progress in this area would be impossible without the development of appropriate screening techniques. Since the frequency of mutant tumor alleles is often less than 0.01% of the total pool of circulating DNA [33,137], the detection methods have to be sufficiently sensitive. In 1999, the droplet digital PCR technology [138] was successfully introduced into practice [139,140,141,142]. The breakdown of the reaction mixture into 20,000 individual droplet microreactors with one target DNA molecule as a matrix allows direct counting of the reaction product when the ratio of the minor allele to the total circulating DNA pool is 1:10,000 (0.01%) [140,143,144]. For that reason, the ddPCR technique is referred as the “golden standard” for quantifying rare mutations [144,145]. Another approach is the establishment of copy number variations (CNVs), a biomarker used for detecting minimal residual breast cancer [52], metastatic breast cancer [53], stomach cancer [54,55], and others [62,143]. Experience gained during the utilization of the ddPCR method has been published in a book by Karlin-Neumann (2018) in the form of detailed protocols [145]. 

In 2010, the Velculescu’s group developed personalized analysis of rearranged ends (PARE) as a method of identifying specific somatic rearrangements in the chromosomal DNA of solid tumors. This method is based on massively parallel sequencing, followed by PCR analysis in order to detect the specific biomarkers in the bloodstream [56,57]. In the study of McBride, the validity of this approach was confirmed through the analysis of more than 100 samples of solid tumors, including cancers of the breast, pancreas, ovaries, bones, and lungs [146]. In all cases, with the exception of one sample, the sites of rearrangements were successfully identified and about 85% of the samples presented more than 10 rearrangements simultaneously. 

Newman and coauthors developed the technology “cancer personalized profiling by deep sequencing” (CAPP-Seq) through the utilization of biotinylated oligonucleotides specific to the most recurrent mutating regions in order to monitor ctDNA in patients with NSCLC [58]. Using this approach, ctDNA was identified in 100% of patients with stage II–IV NSCLC and in 50% of patients with stage I disease with a specificity of 96% and with a frequency of mutant allele fractions of about 0.02% [25,58]. 

A personalized approach based on the genotyping of tumor tissue and the subsequent search of ctDNA in the bloodstream was successfully tested in small pilot studies in bladder urothelial carcinoma [125,147,148]. Later, in the study of Christensen E. (2017), three different NGS-based approaches were used and deletions, insertions, inversions, and intra- and inter-chromosomal translocations were investigated in 337 samples of 12 patients over 20 years [148]. Patel et al. reported that the mutant ctDNA detection in urine by Tam-Seq and genome-wide sequencing could be successfully used for monitoring neoadjuvant therapy effectiveness and clinical prognosis in patients with invasive bladder muscle cancer [60].

A valid support for the identification of new ctDNA biomarkers for the personalized research in cancer may be provided by the analysis of the molecular data already available for several cancers and collected in big consortia like The Cancer Genome Atlas, COSMIC, or GEO Datasets [148,149,150,151,152]. In particular, with the advent of bioinformatics and omics sciences, it is possible to select in silico the putative molecular targets to be analyzed in ctDNA samples, including genetic and epigenetic biomarkers [153,154,155,156]. Alternatively, it might be useful to perform a massively parallel sequencing as done by Leary and coworkers in order to select specific targets to be analyzed in ctDNA [57]. 

In this context, different experimental and computational studies allowed the identification of a specific set of genetic and epigenetics alterations potentially detectable in liquid biopsy samples [157,158]. The investigation of the presence of *BRAF*, *PTEN*, and *KIT* mutations in ctDNA from liquid biopsy samples obtained from melanoma patients was previously described. The analysis of the specific mutations is certainly not accidental. Indeed, these mutations are also analyzed in liquid biopsy samples as they are the most frequently detected mutation in melanoma tumor biopsies, as demonstrated by several studies [159,160]. Other studies have shown that in melanoma patients, the utilization of liquid biopsy samples analysis of other tumor associated factors, including MMP-9 or specific pro-tumoral miRNAs, may represent a useful diagnostic and prognostic approach for the management of this pathology [114,126]. Similarly, different studies showed the usefulness of analyzing the EGFR and KRAS mutations in ctDNA samples obtained from NSCLC patients [84,161].

Colorectal cancer (CRC) patients are often diagnosed when the disease is already advanced or metastatic due to the late onset of tumor symptoms and poor patient compliance to the current diagnostic methods [162]. Hence, a non-invasive, easily accessible diagnostic tool which can detect CRC at an early stage (adenoma) might represent a step forward [163]. For this purpose, numerous studies have studied the potential application of ctDNA detection as a new diagnostic tool. Thankfully, benign tumors and non-neoplastic lesions do not release ctDNA in the circulation making easier the design of a diagnostic ctDNA panel for CRC diagnosis [164]. Until now, only few mutated genes in circulating DNA were confirmed to be valuable as diagnostic tools. For example, the hyper-methylated *SEPT9* gene and the combination of *SEPT9*/*ALX4* mutated genes. Interestingly, the analysis of gene panels compared with single gene mutation tests are best in terms of sensitivity and specificity. For instance, *SEPT9*/*ALX4* panel exhibited a level of 71% sensitivity and 95% specificity for advanced adenomas, thus supporting *SEPT9*/*ALX4* as a biomarker for precancerous lesions [165]. Nonetheless, attempts to achieve an even higher sensitivity and specificity have led to the proposal of a wider gene panel that could include genes such as *HJC1*, *CYCD2*, *PAX5*, *RB1*, *SRBC*, *NPY*, *PENK*, *WIF1*, *ALX4*, *HLFT*, *HPP1*, *MLH1*, *APC*, *CDKN2A*/*P16h*, *TMEFF2*, *NGFR*, *FRP2*, *NEUROG1*, and *RUNX3* [164,166]. Moreover, another panel consisting of methylated *ALU83*, *ALU244*, *OSMR*, and *SFRP1* has also demonstrated its diagnostic value [167]. It is noteworthy that circulating DNA panels have also proved to be useful for prognostic (*APC*, *KRAS*, *TP53*, and *PI3KCA*, methylated *WIF1* and *NPY*), predictive (*APC*, *KRAS*, *TP53*), and therapy tailoring (*KRAS*, *BRAF*, *MET*, *ERBB2*, *FLT3*, *EGFR*, and *MAP2K1*) [168,169]. Accordingly, other independent studies demonstrated that *KRAS* mutation detected in liquid biopsy samples can be proposed as a valid biomarker of diagnosis and prognosis of CRC [170,171]. Other studies confirm the potential diagnostic and prognostic significance of *APC*, *mSEPT9*, and *BRAF* alterations [172,173].

Regarding breast cancer, no conclusive studies have been generated so far. Several studies have tried to propose the evaluation of *HER2*, *PIK3CA* and *PD-L1* expression in ctDNA or circulating tumor cells. However, the specificity and sensitivity values of the analyses were not convincing and the discordance of the data obtained among the molecular subtypes of mammary tumors and among the tumor stages limited the statistical significance of these circulating biomarkers evaluation [174,175,176].

In the following chapters, the analysis of ctDNA diagnostic and prognostic potential will be discussed for the glioma and urothelial cancers. 

Therefore, it is evident that there is no single circulating biomarker for the diagnosis and prognosis of all tumors, but different markers specific for different tumors might exist (Table 3). On this basis, the evaluation of a panel of circulating alterations may provide important information to the clinicians for a correct patient diagnosis and prognosis.

## 6. Circulating DNA in Glioma Diagnosis

Brain tumors are quite difficult to diagnose and are the least treatable. Taking into account the fatal nature of gliomas, the importance of early diagnosis for the patient is of paramount importance [203]. Imaging techniques do not provide comprehensive diagnostic information and biopsy is difficult to obtain. In this case, the use of liquid biopsy is important [204]. The amount of tumor DNA entering the bloodstream in brain tumors is the smallest compared with other tumors, presumably due to the low permeability of the blood brain barrier (BBB) [32]. Importantly, the Alu repeat is one of the most common mobile genetic elements belonging to the short interspersed nuclear element superfamily. It accounts for 10% of the entire genome and is therefore available for research, even with small amounts of ctDNA, as in case of brain pathologies. Alu elements contribute to the development of the disease by two mechanisms: Through insertional mutagenesis and non-allelic homologous recombination, inducing genetic deletions and duplications [205]. Normally, 67.5% of Alu sequences are methylated [50], which hinders their movements in the genome, limiting the level of genetic instability in a healthy cell. A decrease in the methylation level of MGEs increases their motility, causing the genetic instability commonly observed in tumor cells [205,206,207]. Chen et al. (2016) investigated the methylation of plasma Alu repeats in 109 patients with gliomas of four stages of malignancy according to WHO (WHO I–IV) and in 56 patients with benign brain tumors (meningioma and pituitary gland tumor). They used liquid typing on microspheres capable of interacting with 5-hydroxymethylcytosine (hmC) or thymidine in CpG islands (CGIs) of Alu elements following bisulfite conversion. The level of Alu methylation in patients with gliomas was 55.62%. In patients with benign brain tumors, it was 69.16%, and in healthy individuals, it was 67.54%. Differences between patients and controls were statistically significant, and no statistical significance was reported among different types of malignant gliomas. When grouping patients based on mutations in the IDH and TERT genes, the presence of wild-type IDH and mutant TERT in ctDNA (which causes the most unfavorable prognosis) was positively correlated with the lowest Alu methylation levels. The limitation of this work was the prevalence of hypo-Alu-methylation compared with other tumors and physiological conditions, which requires a more focused approach [206]. Nevertheless, the method revealed a significant correlation between the level of Alu methylation and the life expectancy of patients with gliomas, which is vital for prognostic purposes. In addition, regarding tumor stage, Chen et al. showed that liquid biopsy can effectively detect the methylation status of Alu sequences in all cancer stages. In particular, the authors identified lower methylation levels of Alu in grade III–IV advanced gliomas compared to low-grade gliomas (grade I–II) [206]

Faria et al. (2018) [119] determined the changes in circulating DNA concentrations in response to Perillyl alcohol (POH) therapy through the use of a fluorometric method in patients with terminal stage glioblastoma and in patients with brain metastases from stage IV adenocarcinomas localized elsewhere. The patients were compared with a control group of healthy individuals. A dramatic rise in circulating DNA plasma levels was reported in patients with glioblastoma and brain metastases. Such an increase in the concentration of circulating DNA in the bloodstream can be explained by the damage of the BBB present at this stage of the disease [119]. It would be expected that the pre-apoptotic effect of POH would increase the level of apoptotic DNA. However, the authors reported a decrease in circulating DNA levels and an increase in life expectancy, given that the MRI pattern was equivalent to a full response to therapy. It is possible that the level of blood DNAses increases, or the level of their inhibitors decreases, under the influence of anticancer therapy. Discontinuation of the drug treatment caused an increase in the circulating DNA levels. The observed levels of blood circulating DNA are in contrast with the findings of other research groups, which reported very low brain-tumor derived DNA present in the blood (less than 0.01%) [9]. Since the source of circulating DNA is not established in this case due to the use of the fluorimetric method, the origin of circulating DNA remains in question. Nevertheless, in spite of the obscure origin, the amount of circulating DNA can still serve as a marker of the effectiveness of therapy.

As previously mentioned regarding circulating Alu methylated sequences, some studies demonstrated heterogeneity in ctDNA amounts obtained from glioblastoma patients at different tumor stages. More generally, Chetan Bettegowda and coworkers evaluated the concentration of ctDNA in 640 patients with various cancer types, including glioblastoma, and at different stages (metastatic or localized). Overall, the PCR-based methods used showed that >75% of patients with advanced tumors harbor mutant DNA fragments, while ctDNA was found in less than 50% of patients with primary tumors, especially those patients with brain tumors, of which the majority of samples were early-stage tumors [31].

Regarding studies performed exclusively on glioma patients at different stages, Piccioni D.E. and colleagues published a biggest study aimed at evaluating the clinical usefulness of ctDNA analysis. They analyzed 419 primary brain tumors, including 370 astrocytic and oligodendroglial tumors (of which 222 were glioblastoma) [208]. Among the several results obtained, the authors demonstrated that ctDNA mutations rate increased significantly with the more advanced astrocytic and oligodendroglial tumor grades, ranging from 20% of positive grade I samples to 55% of grade IV samples (28% grade II and 40% grade III) [208].

These results confirmed that ctDNA can be more easily identified in higher-grade brain tumors compared to lower-grade tumors. Therefore, ctDNA analysis represents a useful tool for monitoring these patients. Concerning the low-grade tumors, alterations in the circulating DNA are found only in a low percentage of patients. Therefore, the analysis of ctDNA alone may not be sufficient to effectively diagnose tumors at their initial stage. In this case, this analysis must be coupled with the current diagnostics strategies based on imaging techniques.

## 7. Urinary Circulating DNA in Urological Tumors

It was shown that ctDNA was detected in 50% of plasma and in 70% of urine samples from patients with renal cell carcinoma (RCC), prostate cancer (PCa), and bladder carcinoma (BC) [209]. Regarding RCC, ctDNA studies are still in their infancy, but there are many promising roles for both localized and metastatic renal cell carcinoma [210]. ctDNA detection in the plasma of patients with metastatic PCa has paved the way for developing biomarkers, given the impossibility of metastatic tissue sampling. Using liquid biopsies, resistance to androgen receptor (AR)-targeted therapy has been linked to the amplification/mutation of *AR* gene and the expression of truncated splice variants which display ligand-independent activity [47,48,49,211,212,213,214,215]. Therefore, it appears that ctDNA detection is increasingly incorporated into the design of clinical trials with a potential of being integrated into routine patient care. Importantly, ctDNA detection will help to understand PCa development at molecular level and will help lead the way toward a better prognosis and treatment selection, especially with the goal of elucidating tumor resistance mechanisms [216].

In the case of BC, the total pool of circulating DNA in urine was significantly increased in patients compared with the control group [217]. Furthermore, the fragment length of such ctDNA in urine is much greater in patients than in healthy controls [218]. This fact suggests that ctDNA originates from a necrotic tumor, or surrounding tissues that are forced to die under the influence of the tumor-specific molecular signaling [218]. It has been reported that the methylation pattern of *POU4F2* and *PCDH17* in urine ctDNA allows for the discrimination between patients with bladder cancer and patients with other urological conditions compared with healthy volunteers, with 90% sensitivity and 94% specificity [19]. Methylation profiles of *TWIST1* and *NID2* in urine ctDNA allow the identification of patients with a primary tumor with a sensitivity of 90% and a specificity of 93% [219,220,221]. 

Similar to CancerSEEK, a method for screening upper and lower urinary tract urothelial tumors has been developed for large-scale urothelial DNA analysis in urine (called UroSEEK) [222,223]. The test incorporates massive parallel sequencing assays for mutations in 11 genes, including mutations in the *TERT* gene promoter (a hard-to-amplify region, with high GC content), aneuploidy analysis, and copy number changes on 11 gene mutations localized on 39 chromosome arms. This study does not discuss ctDNA, and instead focuses on DNA derived from urothelial cells released in urine samples. However, this method could be easily translated to ctDNA detection. In fact, in a recent study, genomic profiles of urine cell DNA, urine circulating DNA, and DNA from paraffin-embedded samples of 23 well-characterized tumors of patients with BC were compared [13]. The data showed that urine ctDNA patterns correspond with the tumor tissue DNA patterns and have a greater load than the DNA obtained from the urothelium (which is typical for tumor genome) (*P* < 0.001). Consequently, ctDNA has a higher analytical sensitivity for the detection of clinically significant genomic aberrations (*P* < 0.04). It is assumed that the increased tumor genome in ctDNA might be the result of a higher rate of tumor cell necrosis in urine, relative to normal urothelium [13].

As described in the previous paragraph, the study of Bettegowda C. showed an increase of ctDNA levels in high-grade tumors compared to low-grade ones. However, this study contained a limited number of urological samples [31].

Taking into account studies analyzing urological cancers only, Christensen E. and collaborators studied the plasma and urine samples presence of *PIK3CA* and *FGFR3* mutations in more than 800 bladder cancer patients with both non–muscle-invasive bladder cancer (NMIBC) and radical cystectomy following muscle-invasive bladder cancer (MIBC) [148]. As opposite to what has been described for glioblastoma, in this study, the authors showed that patients with low-grade tumors (NMIBC) had a 36% circulating mutation rate compared to patients with high grade-tumors (MIBC), who had a percentage of 11% of ctDNA positivity [148]. These data can be explained because the patients having cystectomies performed right after MIBC diagnosis theoretically do no longer have the tumor mass. Therefore, they cannot release any ctDNA into the bloodstream or urine. However, an interesting reported finding was that patients with cystectomy and positive for *PIK3CA* and *FGFR3* circulating mutations had a higher incidence of tumor recurrence [148]. Therefore, in this case, the liquid biopsy can also provide useful information to the clinician to predict the patient’s prognosis and to identify early tumors which will give rise to recurrence or potentially will evolve from NMIBC to MIBC.

## 8. Multiplex Genotyping Based on ctDNA Mass-Spectrometry

Although, at present, the leading approach for the characterization of ctDNA is the NGS approach, other methods based on mass spectrometry are being developed. Short oligonucleotide mass analysis (SOMA) was proposed in the early 2000s for the study of ctDNA in plasma and in urine [51]. The use of the HPLC protocol ESI-MS-SOMA allowed for the differentiation between patients with hepatocarcinoma and patients with liver cirrhosis or healthy individuals by monitoring the mutation in the 249 codons of the *TP53* gene [51]. The method was not widely used and its application was limited to the characterization of plasma tumor DNA in hepatocarcinoma, although it was characterized as a sensitive technique for detecting mutations. Further developments led to the use of high-resolution mass spectrometry methods with Matrix-assisted laser desorption/ionization time-of-flight-mass spectrometry (MALDI-TOF-MS) [59,224]. The advantage of MALDI is the formation of singly charged oligonucleotide ions, the separation of which depends on the structure of the analyte and the restriction enzyme used. In addition, the advantage of TOF detection is the ability to measure masses in excess of 100 kDa. Sequencing is performed by calculating the mass difference between adjacent signals, and the interpretation of the spectra of multicomponent samples is not difficult. Comparison of the signal intensity between the four mass spectra allows the determination of the initial sequence [59]. For that features, MALDI-TOF-MS enables multiplex genotyping and is considered a sensitive, reliable, fast, and cost-effective technology for detecting target mutations in NSCLC patients [225]. MS analysis was used to test 158 mutations of major *EGFR*, *KRAS*, *BRAF*, *ALK*, *PIK3CA*, *ERBB2*, *DDR2*, *AKT*, and *MEK1* oncogenes in 92 NSCLC tumor samples and in 13 plasma samples. Also, cytological samples have been analyzed. Those samples are often a unique source of the original tumor material, but are of poorer quality for different molecular studies. It was observed that a mutated allele with a frequency higher than 10%, was positively detected even with a small amount of DNA (5 ng). With less than 10% of mutated allele, the detection must be performed with 10ng of DNA as starting material. The authors do not recommend the use of molecular diagnostics for the analysis of low-quality samples, with less than 20% of tumor within the whole sample. MS is recommended as an alternative high-performance method for detecting known mutations, even in the case of low-quality samples. It allows genotyping the deficient component of ctDNA in a tissue sample, which has an important impact on the clinical management of the patient [225].

## 9. Limitations of the Use of ctDNA in Cancer Routine Practice

With the progressive development of methods for studying extracellular tumor DNA, the wide clinical application of this technology will occur in a matter of time. The main drawback is genetic heterogeneity and high mutagenesis of tumors [226]. During disease progression, individual disseminated tumor cells and metastases may acquire characteristics different from those of primary tumors [32]. Moreover, although in some cases (melanomas) the mutational status is strictly associated with the anatomical type and localization of the tumor [227], many mutations are secondary and they may be lost by tumor clones during tumor recurrence. Phenotypically similar tumors may include quite different molecular genotypes, representing the individuality of each tumor and each patient [228]. The dynamic and heterogeneous nature of the tumor mass is characterized by pronounced genomic instability, somatic mutations, and epigenetic changes and, despite the advancement of surgical, radiological and pharmacological treatment, is worsened following cytotoxic chemotherapy and ionizing radiation therapy [66,119,229]. It still remains under question whether a mutational status might represent a valid basis for the selection a personalized type of therapy (oncogenic driver mutations) or whether it might be used only as a diagnostic tool [230]. Consensus at the Food and Drug Administration (FDA) level has only been reached for the treatment of tumors with certain mutations of the DNA repair systems (and, as a result, microsatellite instability), coupled with pembrolizumab (checkpoint blockade) based-therapy. This is the first case where it is not the anatomical location of a tumor, but its genetic features to dictate the selected immunotherapy to be chosen for the patient [231]. 

The limitations of the ctDNA approach are summarized in the comments of various authors concerning CancerSEEK methodology:

- The majority of patients in the study had stage II–III of disease, which does not yet provide an opportunity to assess the applicability of the test for early diagnosis [232].
- As control, the authors analyzed the urine of only healthy individuals. Thus, the high specificity of the CancerSeek approach requires further validation with non-cancer control with associated diseases, such as inflammatory diseases of the genitourinary system, which are common in the elderly [233].
- The limitations of the method are linked with the same heterogeneity of tumors, such as lung cancer [234]. These circumstances cannot be attributed to the shortcomings of the method, but make it necessary to add additional markers that may increase the accuracy of the study, such as RNA of exosomes for early diagnosis of non-small cell lung cancer [235].

The authors themselves state that the CancerSEEK does not work equally for all cancers. The panel achieved a total median sensitivity of 70% with a specificity of ≥99%, but significant differences in sensitivity were observed between the different types of tumors analyzed (e.g., 98% for ovarian cancer, 60% for lung cancer, and only 33% for breast cancer) [36]. 

In general, most of the available studies cover narrow areas of the wide oncology field, they analyze only specific types of tumors and they need to be confirmed on larger patient cohorts.

## 10. Conclusions

Used as primary oncologic screening, liquid biopsy represents a valid alternative to tissue biopsy which allows the clinicians to focus on the most common, early, and stable markers, coupled with a specific the oncologic process. Such liquid biopsy allows doctors to perform the therapeutic profiling of cancer patients to monitor the anticancer therapy efficacy, as well as to predict the progression of an already identified cancer. In this way, it is possible to identify fine alterations of markers specific for each patient [35]. 

This review can be summarized with the words of Bert Vogelstein, MD, Clayton Professor of Oncology and Howard Hughes Medical Institute researcher: “If we are going to make progress in early cancer detection, we have to begin looking at it in a more realistic way, recognizing that no test will detect all cancers” [236].

## Figures and Tables

**Table 1:** Liquid biopsy versus tissue biopsy.

Characteristics and Scope	Liquid Biopsy	Tissue Biopsy
Invasiveness	Minimally invasive	Invasive
Study prescription time	On demand repeatedly	Prior a therapy prescription
Sample degradation	No, long keeping in −70 °C [35]	Cross-linking and DNA fragmentation [35]
Tumor size/number of tumor cells for detectable ctDNA	5 × 10^7^ cells [9]	>10^9^ cells [9]
Amount of biomaterial	3 mL peripheral venous blood	Depending on the technique and organ
Screening	Yes [36]	No
Therapy choice	Yes	Yes
Continuous dynamic observation (monitoring)	Yes, half-life of ctDNA between 16 min and 13 h [32,33,34]	No, too traumatic
Response to therapy	Yes [21,34]	No
Residual tumors	Yes [21,34]	No
Relapse prognosis	Yes [21,34]	No

**Table 2:** Tumor circulating DNA blood tests in personalized cancer diagnostics and cancer characterization.

Purpose of Research	Method	Example of Application	Specimen	Sensitivity Specificity %
Early diagnostics, screening	Target Deep Sequencing -TamSeq	Ovaries cancer (*EGFR*, *TP53* *) [45]	Plasma	>97/>97
	Target Deep Sequencing -Ion-AmpliSeq (Ion Torrent)	Breast cancer (*p53, PIK3CA, PTEN, AKT1, IDH2, SMAD4*) [27]	Plasma	n/a
	Target Deep Sequencing by Illumina- Hi-Seq	Pancreatobiliary Carcinomas (*KRAS, TP53, APC, FBXW7, SMAD4*) [46]	Plasma	>92/100
	Massively Parallel Sequencing - CancerSEEK	Eight types of cancer (ovaries, liver, stomach, pancreas, esophagus, rectum, lungs and breast) [36]	Plasma	On average 70/>99
	Quantitative Methylation Specific PCR	BC (*POU4F2 и PCDH17*)^7^; BC (*TWIST1 и NID2*) [47,48,49]	Urine	90/94 90/93
	The liquid typing on microspheres	Gliomas WHO I-IV (The level of Alu methylation) [50]	Plasma	n/a
	HPLC ESI-MS-SOMA	HCC (p53) [51]	Plasma	n/a
Identification of cancers of unknown primary	Target Deep Sequencing by Illumina- Hi-Seq or Next-Seq	SCLC, squamous cell lung cancer, colorectal adenocarcinoma, HCC and duct carcinoma of the mammary gland in situ [22]	Plasma	n/a
Detection of minimal residual tumor	ddPCR-CNVs	Breast cancer (*USP17L2 (DUB3), BRF1, MTA1*, and *JAG2*) [52]	Plasma	n/a
Metastasis detection	ddPCR-CNVs	Breast cancer [53] Gastric cancer (*MET*, *HER2*) [54,55]	Plasma	93/100 73.3/93.3
	Target Deep Sequencing -TamSeq	Breast cancer (*PIK3CA*, *TP53*) [37]	Plasma	n/a
Integral tumor profiling in each specific patient	Massively Parallel Sequencing-PARE + PCR	Specific somatic rearrangements in the chromosomal DNA of solid tumors and plasma ** [56,57]	Tumor tissue + Plasma	n/a
	Target Deep Sequencing - CAPP-Seq	NSCLC [25,58]	Plasma	85/96 ***
	MALDI-TOF-MS	NSCLC (*EGFR, KRAS, BRAF, ALK, PIK3CA, ERBB2, DDR2, AKT*, and *MEK1*) [59]	Tumor tissue + Plasma	n/a
Monitoring of therapy effectiveness and clinical prognosis	Target Deep Sequencing -Tam-Seq and genome-wide sequencing	Invasive bladder muscle cancer [60]	Urine	83/100
Diagnostics, screening, monitoring etc	ddPCR-SNP and chromosome rearrangement	Different tumor types (*PIK3CA KRAS, BRAF, NRAS*, and *EGFR*) ** [31,61,62]	Plasma	87.2/99.2

* Allele frequency 2%. ** Allele frequency 0.01%. *** Allele frequency 0.02%. Abbreviations: NGS—next generation sequencing; TAmSeq—tagged-amplicon deep sequencing; Hi-Seq—high-performance sequencing; HPLC ESI-MS-SOMA—short oligonucleotide mass analysis; ddPCR—droplet digital PCR; CNVs—copy number variation. PARE—personalized analysis of rearranged ends; MALDI—TOF-MS—matrix-assisted laser desorption/ionization time-of-flight-mass spectrometry; BC—bladder cancer; WHO—World Health Organization; SCLC—small cell lung cancer; NSCLC—non-small cell lung cancer; HCC—hepatocellular carcinoma.

**Table 3:** Overview of the most relevant ctDNA and CTCs analyses in different cancer types.

Molecular Target	Sample Type	Technology	Study(ies)
**Non-Small Cell Lung Carcinoma**			
*EGFR* mutation	ctDNA	ddPCR	[177]
	ctDNA	ddPCR	[178]
	CTCs	NGS	[179]
*PD-L1* expression	CTCs	Immunofluorescence	[180]
	ctRNA	qPCR	[175]
	CTCs	Immunofluorescence	[181]
**Colorectal Cancer**			
*APC* mutation	ctDNA	NGS	[172]
	CTCs	NGS	[182]
	ctDNA	ddPCR	[183]
*KRAS* mutation	ctDNA	COLD PCR, Microarray, ddPCR	[184]
	ctDNA	NGS	[185]
	ctDNA	ddPCR	[139]
	CTCs	Nested PCR	[186]
*BRAF* mutation	ctDNA	PCR-Microarray	[187]
	ctDNA	ddPCR	[188]
	CTCs	qPCR	[189]
*mSEPT9* methylation	ctDNA	Real-Time PCR	[190]
**Breast Cancer**			
*HER2* expression	ctDNA	NGS	[191]
	CTCs	Immunofluorescence	[192]
*PD-L1* expression	ctRNA	qPCR	[175]
	CTCs	Western blot Flow Cytometry Immunocytochemistry	[193]
*PIK3CA* mutation	ctDNA	ddPCR	[176]
	ctDNA	NGS	[194]
	ctDNA	NGS	[195]
	CTCs	ddPCR	[134]
**Cutaneous Melanoma**			
*BRAF* mutation	ctDNA	ddPCR	[126]
	CTCs	ddPCR	[196]
	ctDNA	Exome NGS	[197]
*PTEN* mutation	ctDNA	SNPase-ARMS qPCR	[198]
	CTCs	NGS RNA	[199]
*TERT* promoter mutation	ctDNA	ddPCR	[200]
*KIT* mutation	ctDNA	NGS	[201]
	CTCs	hemi-nested PCR	[202]

## Supplementary Materials

The following are available online at https://www.mdpi.com/2073-4409/8/10/1251/s1.
