# Supplementary material for: Translational Application of Circulating DNA in Oncology: Review of the Last Decades Achievements
Source: Cells. 2019 Oct 14;8(10):1251. doi: 10.3390/cells8101251 (PMC6829588; doi:10.3390/cells8101251)
Supplement: Supplementary file 1 [file cells-08-01251-s001.pdf]

Supplementary Table S1. GRAIL Clinical Research Program (2016 – 2020)

Official Title	Participants	Characteristics of the study	NCT number *	Current Status
CCGA Study: Circulating Cell-free Genome Atlas Study	15,000 (men and women age after 20 years)	Prospective, observational, longitudinal, study designed to characterize the landscape of genomic cancer signals in the blood of people with and without cancer	NCT02889978	Follow-up
The STRIVE Study: Breast Cancer Screening Cohort for the Development of Assays for Early Cancer Detection	99,481 (women)	Prospective, observational, longitudinal, cohort study that has enrolled approximately 100,000 women at the time of their screening mammogram	NCT03085888	Follow-up
SUMMIT Study: Evaluation of a New Blood Test for Detecting Multiple Types of Cancer Early	50,000 (men and women age 50 to 77 years)	prospective, observational, longitudinal, cohort study to evaluate a blood test designed to detect multiple types of cancer, including lung cancer	-	Enrolling

* https://www.clinicaltrials.gov
